# Distributed Fiber Optical Sensing of Oxygen with Optical Time Domain Reflectometry

**DOI:** 10.3390/s130607170

**Published:** 2013-05-31

**Authors:** Susanne Eich, Elmar Schmälzlin, Hans-Gerd Löhmannsröben

**Affiliations:** 1 Institute of Chemistry/Physical Chemistry, University of Potsdam, Karl-Liebknecht-Str. 24-25, Potsdam-Golm 14476, Germany; E-Mail: loeh@chem.uni-potsdam.de; 2 Colibri Photonics GmbH, Am Mühlenberg 11, Potsdam-Golm 14476, Germany; E-Mail: schmaelzlin@colibri-photonics.com

**Keywords:** OTDR, optical sensing, molecular oxygen, triangular-[4] phenylene

## Abstract

In many biological and environmental applications spatially resolved sensing of molecular oxygen is desirable. A powerful tool for distributed measurements is optical time domain reflectometry (OTDR) which is often used in the field of telecommunications. We combine this technique with a novel optical oxygen sensor dye, triangular-[4] phenylene (TP), immobilized in a polymer matrix. The TP luminescence decay time is 86 ns. The short decay time of the sensor dye is suitable to achieve a spatial resolution of some meters. In this paper we present the development and characterization of a reflectometer in the UV range of the electromagnetic spectrum as well as optical oxygen sensing with different fiber arrangements.

## 1. Introduction

Fiber optical chemical sensors are commonly based on absorption or fluorescence of the analyte or a sensor dye which interacts with the analyte [1]. Using fibers for chemical sensing allows remote measurements in environments, which are difficult to access. The technique of optical time domain reflectometry (OTDR) has the potential to further improve fiber optical chemical sensing because only one end of the fiber is needed for measuring. Furthermore, it is possible to obtain signals of sensors at different positions resulting in spatial resolution along the fiber which is relevant for some applications, like in piscicultures [2] and in cell bioreactors [3]. The combination of OTDR with fiber optical chemical sensors is a promising approach for distributed fiber optical chemical sensing because it is a parallel, scalable and spatially sensitive method.

Oxygen is one important analyte because of the outstanding role in many biological and technical processes. Consequently, the determination of oxygen concentrations is of very high importance in life science, biotechnology, medicine and industrial processes. Optical oxygen sensing is an established method in the field of fiber optical chemical sensing. We combine this method with the technique of OTDR to enable distributed measurements.

### 1.1. Optical Sensing of Oxygen

Non-optical methods, like Clark electrode based measurements via the reduction of oxygen at the cathode, suffer from one major drawback: the consumption of the analyte. In contrast, optical oxygen sensing is based on luminescence quenching of a sensor dye, resulting in a decrease of the luminescence intensity and/or decay time. In practice, it is favorable to quantify the decay times instead of intensities, since decay times are largely independent of light source fluctuations, dye concentration, as well as scattering and absorption within the light path. Dynamic luminescence quenching can be described by the Stern-Volmer Equation (1):

(1) $$\frac{\tau_{0}}{\tau }=1+K_{\text{SV}}\times [{\text{O}}_{2}]=1+k_{\text{q}}\tau_{0}\times [{\text{O}}_{2}]$$

Herein, τ_0_ and τ are the luminescence decay times in absence and in presence of oxygen, respectively; [O_2_] is the oxygen concentration, *K*_SV_ the Stern-Volmer constant, and *k*_q_ the quenching rate constant. With the help of the uncertainty of the blank sample σ and the Stern-Volmer constant the limit of detection (*LOD*) can be calculated:

(2) $$\text{LOD}=\frac{3\sigma }{K_{\text{SV}}}$$

Common oxygen sensor probes are metal porphyrins incorporated in a solid polymer matrix [4,5], doped polymer nanobeads [6] or covalently bonded on polymer chains [7]. The luminescence decay times of these metal porphyrins in absence of oxygen is mostly longer than 1 μs. Another class of molecules, which are suitable for oxygen sensing are polycyclic aromatic hydrocarbons (PAH), especially pyrene and perylene [8]. The decay time of these compounds is much shorter than the luminescence decay time of metal porphyrins (for perylene: τ_0_ = 6.4 ns) [9].

Nevertheless, the very long (μs) as well as the very short (few ns) luminescence decay times of the conventional oxygen sensor dyes disqualify them for the combination with optical time domain reflectometry (OTDR) as will be explained below.

### 1.2. Distributed Sensing with OTDR Technique

Optical time domain reflectometry (OTDR) is a well known technique in optical fiber communication and was first demonstrated by Barnoski and Jensen [10]. Initially, a laser pulse is launched into a fiber. Absorption and scattering of the laser light leads to an exponential decrease of the pulse power *P* along the fiber (Equation (2)):

(3) $$P(l)=P_{0}\times \exp(-\alpha l)$$

*P*_0_ is the initial pulse power, *l* is the fiber length and *α* (in 1/m) is the total attenuation coefficient. A small part of the scattered light is guided back to the beginning of the fiber and is detected time-dependently. With the knowledge of the group index of the fiber the time scale can be converted into a length scale.

The backscattered power *P*_bsc_ can be calculated with Equation (3) [11]:

(4) $$P_{\text{bsc}}(l)=\frac{1}{2}S\alpha_{\text{R}}P_{0}T_{0}c_{\text{g}}\times \exp(-2\alpha l)$$

*T*_0_ is the pulse width of the laser pulse, *c*_g_ is the group velocity of light in the fiber, *α*_R_ is the attenuation coefficient caused by Rayleigh scattering, and *S* is the recapture factor and corresponds to the part of the scattered light which is guided back. *S* depends on the geometrical architecture of the fiber, and for a multi-mode step index fiber *S* is [12]:

(5) $$S=\frac{3}{8}\frac{NA^{2}}{n_{1}^{2}}$$

where *NA* is the numerical aperture of the fiber and *n*_1_ is the refractive index of the fiber core.

The common logarithmic ratio of *P*_bsc_ and *P*_0_ leads to the attenuation *a* in dB and the corresponding attenuation coefficient *α*_dB_ (Equation (5)). If plotted against the fiber length *l*, *a* contains spatially resolved information about fiber characteristics like *α*_dB_ and losses at splices and connectors:

(6) $$\begin{array}{cc} a(l)=10\times \mathrm{lg}\left(\frac{P_{0}}{P_{\text{bsc}}(l)}\right)\text{dB} & \alpha_{\text{dB}}=\frac{a(l)}{L}(\text{dB}/\text{m}) \end{array}$$

In general there are two important parameters to characterize a reflectometer, dynamic range *(DR)* and the spatial resolution. The *DR* in dB is the difference between the measured attenuation *a* at the beginning of the fiber and the background (in OTDR literature often referred to as “noise floor”). The *DR* depends strongly on backscattered power, the sensitivity of the detector and the number of repeated measurements, and it can be calculated with the “*OTDR maker's formula*” [13]:

(7) $$DR(\text{dB})=P_{\text{init}}-2\alpha_{\text{dB}}l-P_{\text{NEP}}+1.5\log_{2}N$$

*DR* is the dynamic range ratio after *N* measurements, *P*_init_ reflects the initial backscattered power at the beginning of the fiber, *P*_NEP_ is the noise equivalent power of the detector and *N* the number of measurements. The unit of *P*_init_ and *P*_NEP_ is dBm. When decreasing *P*_NEP_ and increasing the number of measurements the dynamic range increases. With Equation (6) it can be shown that in order to reach a *DR* of 30 dB the number of measurements should be in the range of 10 [7]. Hence, using a laser with a repetition rate of 1 kHz will result in a measuring time of about 10 min. Common reflectometers with an operating wavelength of 630 nm for multimode fibers reach a *DR* of around 35 dB [14].

The two point resolution of a reflectometer is the minimal fiber length Δ*l* between two dispersive events that can be resolved by the detector. It strongly depends on the pulse width *T*_0_ of the laser (Equation (7)):

(8) $$\Delta l=\frac{1}{2}c_{g}T_{0}$$

In the case of reflections or sensoric events along the fiber line the two point resolution is no longer the key parameter for the spatial resolution. The amount of light which travels back following a reflection leads to signal saturation at the detector, which in turn needs time to recover. During this time no other event can be detected, thus limiting the spatial resolution of a reflectometer. In the case of sensor applications, the luminescence decay time of the sensor dye increases the period of time, in which no other event can be detected, thereby further reducing the spatial resolution of the reflectometer.

To take the additional effects into account, the two point resolution is replaced by the dead zone, which can be differentiated in event dead zone and attenuation dead zone. The event dead zone is defined as the minimum fiber length, which is needed to detect two close-by events. The time between the beginning of the event and the moment where the reflected peak has lost 1.5 dB is measured, resulting in a distance, which is known as the event dead zone.

The attenuation dead zone is the minimum distance, which is required for a complete separation of two consecutive events. It is defined as the length from the start of a reflective or sensoric event and the point where the backscatter trace returns to within 0.1 dB of the backscattered level. Again this is measured in units of time and converted to a length.

In principle, there exist two possibilities to determine the typical reflector characteristics like *DR* and dead zone, either by calculations with the formulas mentioned above or with the help of measured data. Both approaches will be presented in this paper.

Sensor application of the OTDR principle for physical parameters like strain and temperature are well known [15,16]. Chemical OTDR sensing based on changes of absorption [11,17,18], of refractive index [19] and also luminescence intensity [20] were reported. In literature the combination of OTDR with life time based luminescence sensor dyes is mentioned [21], but to the best of our knowledge not yet realized.

Common fluorescence probes for oxygen sensing show decay times in the micro- and millisecond range [2,3,22], which is experimentally convenient for classical luminescence spectroscopy. However, for OTDR, sensing probes with microsecond lifetimes are not suitable, since they would increase the attenuation dead zone to some kilometers. This disqualifies the commonly used oxygen sensitive metal porphyrins as sensor dyes. On the other hand, the short decay time of perylene demands expensive experimental equipment. Therefore, a novel oxygen sensor dye with a luminescence lifetime in the 10–100 ns time range is required. Triangular-[4]phenylene (TP) shows a luminescence lifetime between 80 ns in absence and 20 ns in presence of oxygen [23] making it an ideal sensor dye for oxygen sensing using the OTDR principle. This dye shows a moderate absorption at 350 nm (absorption coefficient ε_350nm_ ≈ 15,000·M^−1^cm^−1^, in THF) [23] and a bright fluorescence between 400 nm and 550 nm (fluorescence quantum yield Φ_F_ = 0.15, in THF) [23]. Commercially available reflectometers have an operating wavelength between 630 nm and 1,550 nm. It was therefore necessary to build a reflectometer, which is suitable for OTDR oxygen sensing within the UV range of the electromagnetic spectrum. In a previous work [24] we used TP dissolved in toluene as sensor dye with the OTDR technique and proved the feasibility of this sensor dye for oxygen sensing with OTDR. Since dye solutions are unpractical for sensor applications it is necessary to incorporate the sensor dye in a solid matrix. This matrix must fulfill some requirements, like transparency at the excitation wavelength of the sensor dye as well as permeability for oxygen. In this paper TP immobilized in a silicone matrix is used for the first time as an OTDR oxygen probe, and the capability of OTDR for distributed oxygen measurements is demonstrated.

## 2. Experimental Section

**Figure 1.**
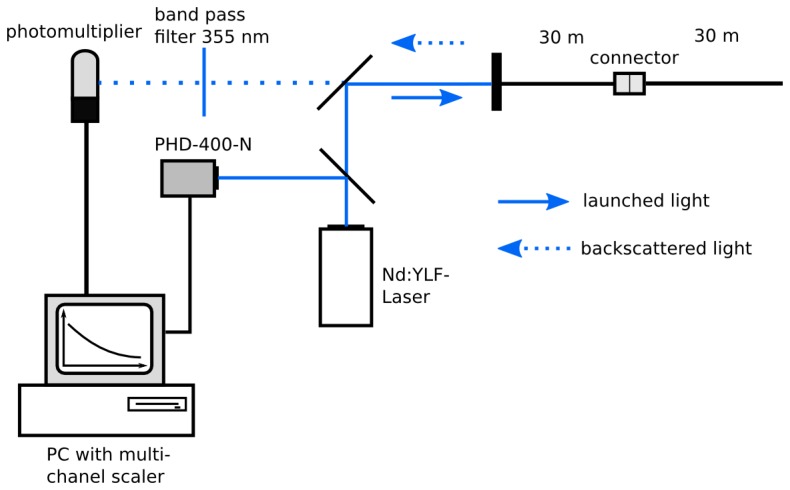
Optical setup of the reflectometer and fiber arrangement for the determination of parameters of the reflectometer; laser light passes a beam splitter for triggering the measurements and launches through a second beam splitter and collimator into two 30 m long connected fibers. Scattered light passes the second beam splitter, a band pass filter and is measured with a photomultiplier. Data are recorded with a multichannel scaler card.

The optical setup of our UV-reflectometer is shown in Figure 1. A diode-pumped, Q-switched, frequency tripled Nd-YLF-Laser (*Explorer*, Spectra Physics, Mountain View, CA, USA) with an excitation wavelength of 355 nm and 2.5 kHz repetition rate (pulse width: 5 ns; pulse energy: 25 μJ ) was used as light source.

To trigger the data acquisition, a fraction of the laser beam was guided to a photodiode (PHD-400-N, Becker and Hickl, Berlin, Germany). The excitation signal was deflected by a beam splitter and launched into the fiber network. The light backscattered from the fiber was guided through the fiber network, passed the beam splitter, a (355 ± 5) nm band pass filter, and was detected by a single photon photomultiplier module (PMC100-1, Becker and Hickl). To guide the backscattered light to the detector, two mirrors were used. Signal intensities were recorded by a P7889 Multi-Channel-Scaler Card (FAST ComTec, Oberhaching, Germany) as a function of time with a resolution of 0.1 ns and a number of measurements of *N* = 500,000. Several neutral density filters (transmission 0.5%, 7% and 24%) were used to attenuate the laser light for decreasing the intensity of reflections and avoiding saturation of the detector.

To determine the dynamic range, dead zone and spatial resolution of the UV-reflectometer a simple fiber arrangement of two connected 30 m fibers was used.

**Figure 2.**
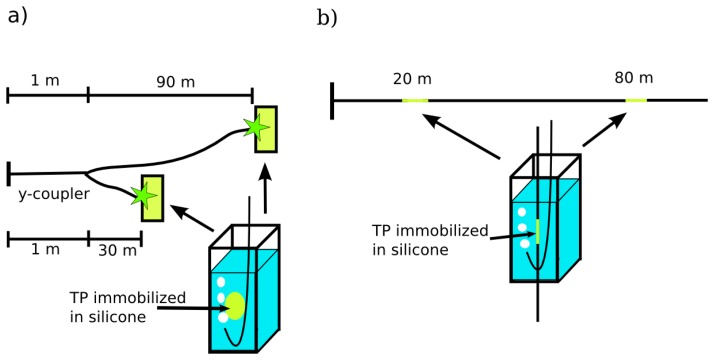
(**a**) Fiber arrangement of the branched fiber model. (**b**) Fiber arrangement of the single linear fiber model.

For oxygen sensing the luminescence light has to be separated from backscattered excitation light. Therefore the beam splitter was replaced by a dichroic mirror and the (355 ± 5) nm band pass filter was substituted by a (480 ± 10) nm band pass filter. In general there are two possibilities for distributed fiber optical sensing: A branched fiber arrangement where the sensor spots are at the ends of each branch, and a single linear fiber model for evanescent wave application where the sensor dye is immobilized around the optical fiber. Therefore, both different fiber arrangements were investigated: Firstly, a branched fiber model with two fibers (30 m and 90 m) that are connected to a home-made y-coupler and possess a solid sensor spot at the end of each fiber (Figure 2(a)). Secondly, a single fiber with two sensor points at 20 m and 80 m for sensing using evanescent wave interactions (Figure 2(b)). The fiber was tapered at the sensor positions and a solid sensor film was wrapped around these tapers. In both fiber arrangements the sensors were placed in PMMA-cuvettes filled with water.

The gas mixtures were prepared with digital mass flow controllers (MFC, Brooks, Hatfield, PA, USA). For the blank sample, the water was deaerated with pure nitrogen. The oxygen sensing measurements were performed at room temperature with no further control.

All used fibers were 200/220 quartz/quartz fibers (NA = 0.22, *α*_355 nm_ = 220 dB/km, *α*_480 nm_ = 35 dB/km, FiberTech GmbH, Berlin Germany). The y-coupler was home-made by fusion splicing of a 400/420 quartz/quartz fiber with two 200/220 quartz/quartz fibers (both fibers NA = 0.22, FiberTech GmbH).

Syntheses, chemical, optical and electronic properties of the TP sensor dye were described previously by Dosche *et al* [23]. For the preparation of the immobilized sensor spots silicone (Silastic 734 RTV) was spread on a glass slide and cured for 24 h at room temperature. Afterwards, the solid silicone was placed in 0.08 mM (first sensor point) and 0.8 mM (second sensor point) TP solutions in toluene for 24 h, removed, washed with toluene and dried for 1 h at room temperature. Different TP concentrations were used for adjusting the intensity of the fluorescence signals with the higher concentration for the sensor points at the longer distances. Photophysical characterization of the solid sensor spots was performed with the spectrofluorometer Fluoromax4 with a TCSPC module (HORIBA Jobin Yvon GmbH, Unterhaching, Germany).

## 3. Results and Discussion

### 3.1. Characterization of UV-Reflectometer

**Figure 3.**
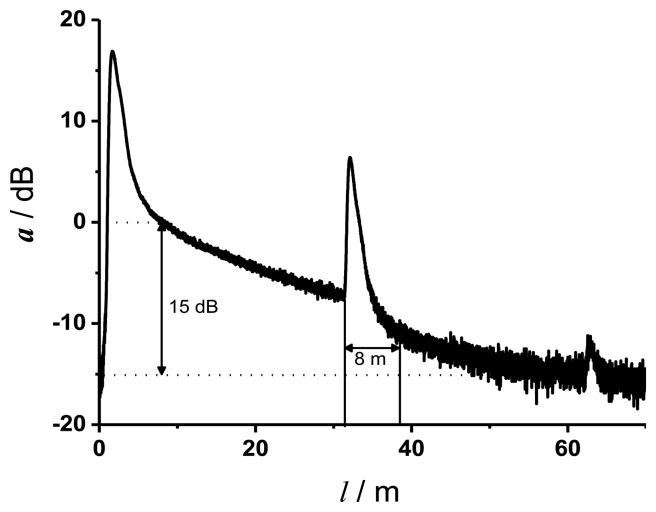
Converted OTDR trace (attenuation *versus* fiber length). The parameters determined with these data are: dynamic range = 15 dB, attenuation dead zone = 8 m.

Figure 3 shows a typical OTDR trace (attenuation *a* in dB *versus* fiber length *l* in m) at the excitation wavelength of 355 nm of two approximately 30 m long fibers, which were connected by a SMA connector. The peaks represent the reflections at the beginning, at the connector and the end of the fiber line. The linear slope between the first reflection and the reflection at the connector represents the attenuation coefficient *α*_dB_ of the fiber material.

Theoretically, a dynamic range of the home made reflectometer of 20 dB is achieved. This is in good agreement with the dynamic range of 15 dB determined from Figure 3. The lower value of 15 dB is due to the SMA connector and the optical components, which were needed to couple the backscattered and reflected light to the detector. An event dead zone of 1.8 m and an attenuation dead zone of 8 m were determined. Graphical determination of the parameters was conducted according to the definitions in the IEC document [25]. From Figure 3 it is obvious that the maximal fiber length for measurements with a wavelength of 355 nm is limited to about 60 m because of the high attenuation coefficient at 355 nm.

Sensoric elements included along the fiber line lead to an increase of the maximal fiber length because of the smaller attenuation coefficient of the fluorescence emission light, which travels in backward direction to the detector. On the other hand fluorescence sensor dyes along the fiber line lead to an increase of the attenuation dead zone, which depends on the luminescence decay time of the sensor. For an exponential decay the emission signal vanishes completely after about ten times the luminescence decay time. In the case of TP in the absence of oxygen, the sensor decay time is in the range of 80 ns leaving no measurable luminescence signal after approximately 800 ns. According to the group index of the fiber material, the attenuation dead zone should therefore be not longer than 80 m. This increase of the attenuation dead zone, and finally the decrease of the spatial resolution, indicates the limitations of sensor applications with the OTDR principle. In the range of the dead zone (=decay curve of the sensor dye) no other sensor point can be included into the fiber arrangement, otherwise an exact data evaluation of the decay curves becomes impossible.

### 3.2. Characterization of Solid Sensor Spot

**Figure 4.**
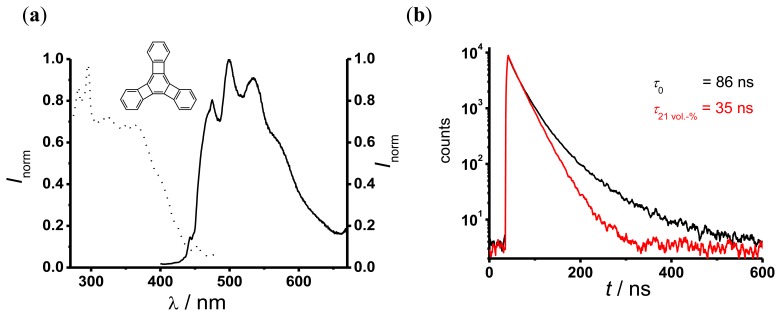
Excitation spectra (dotted line; *λ*_em_ = 500 nm) and emission spectra (solid line; *λ*_ex_ = 350 nm) of TP in silicone (**a**); fluorescence decay curves at different oxygen concentration, τ_0_ = 86 ns; τ_21vol.-%_ = 35 ns (**b**).

Figure 4(a) shows the fluorescence excitation (dotted curve) and emission (solid curve) spectra of TP-doped silicone. The fluorescence excitation spectrum shows a moderate absorption around 355 nm, which is the operating wavelength of the UV reflectometer. The fluorescence emission spectrum shows a bright fluorescence between 450 nm and 600 nm. The decay curves of TP in silicone (Figure 4(b)) indicate a multi-exponential behavior. Therefore, to evaluate the oxygen dependent decay time a dual-exponential decay function (Equation (8)) was used:

(9) $$I=I_{0}+A_{1}\times \exp(-t/\tau_{1})+A_{2}\times \exp(-t/\tau_{2})$$

*I* is the fluorescence signal intensity, *I*_0_ the background intensity, *A*_1_ and *A*_2_ are the amplitudes and τ_1_ and τ_2_ are the TP decay times in this sensor. All fits to the measured data with Equation (8) yielded similar values of an oxygen independent component (τ_1_ ~ 15 ns, *A*_1_ between 0.1–0.5) and an oxygen dependent component τ_2_ (in the following simply referred to as τ). The values of τ were determined by performing a global fit with τ_1_ as a shared parameter. This procedure yielded, e.g., τ_0_ = τ (0 vol.-% O_2_) = 86 ns and τ (21 vol.-% O_2_) = 35 ns. Further experiments showed no influence of chloride anions up to a concentration of 0.2 M on the oxygen dependent decay times.

The response time *t*_90_ of the solid sensor system is an important parameter for sensor characterization and is the time, which is needed to reach 90% of the sensor signal. Short response times are desirable for many applications. Response times of some seconds for transition metal based oxygen sensor probes in different matrices are reported [3].

**Figure 5.**
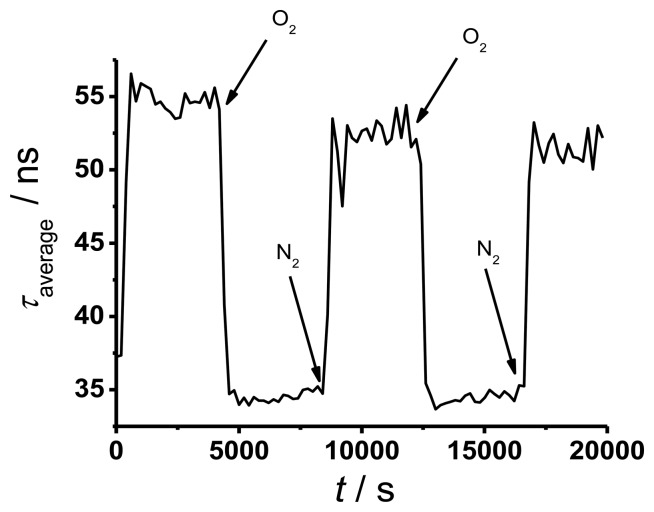
Time response of TP in silicone.

To determine the response time of TP in silicone, a solid sensor spot (in a cuvette filled with water) was placed at the end of a 50 m long fiber and the decay curve was measured continuously while changing the oxygen partial pressure from 220 mbar to 0 mbar and *vice versa* for three times. The result is shown in Figure 5 where the averaged decay times of the sensor point exposed to different atmospheres were plotted *versus* time.

The decay times in Figure 5 show good reversibility with some evidence of photo degeneration. This effect is small and of less importance for oxygen sensing. The determined response time for the solid sensor spot is around 6 min. This value is long compared to literature data [3], but it is sufficient for many future applications.

### 3.3. Quasi-Distributed Oxygen Sensing

**Figure 6.**
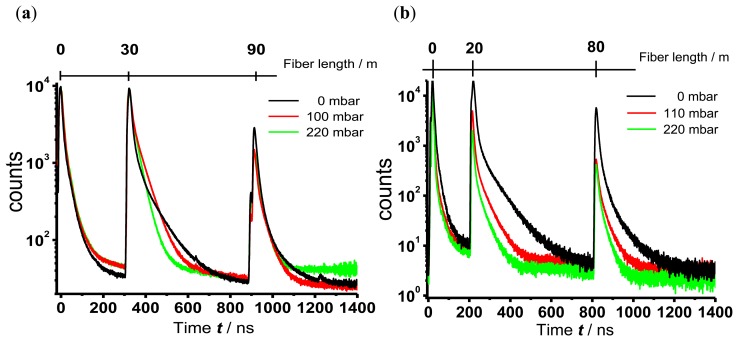
OTDR traces of different oxygen concentrations of (**a**) the branched fiber model (**b**) the single linear fiber model for the evanescence wave application.

In Figure 6 OTDR sensor traces measured with the branched fiber model (Figure 6(a)) and the single linear fiber model for evanescence wave application (Figure 6(b)) of TP immobilized in silicone are shown. For clarity only three different partial pressures of oxygen, 0 mbar, 110 mbar and 220 mbar (corresponding to 0 vol.-%, 11 vol.-% and 22 vol.-% oxygen in nitrogen), are displayed. In the case of evanescence wave sensing the area of interaction between the excitation light and the sensor dye is nearly ten times larger than the area of interaction in the branched fiber model, where this area is limited through the diameter of the fiber end. The large area in case of the evanescent wave sensing leads to a high coupling efficiency of fluorescence light into the fiber, thus increasing the intensity of the signal. To avoid signal saturation of the photo multiplier the gain voltage was adjusted leading to a lower noise in the OTDR traces of the evanescence wave sensing.

The signal peak at the beginning of the trace derives from fluorescence light of the optical components that are needed to launch the excitation pulse into the fiber. The sensor peaks are well separated (branched fiber model: 300 ns and 900 ns, corresponding to 30 m and 90 m; single fiber model: 200 ns and 800 ns, corresponding to 20 m and 80 m). Therefore, the previous considerations about the attenuation dead zone of the sensor signal and the maximal length of the fiber with included sensor points are confirmed.

As expected, an oxygen dependent decrease of the luminescence decay times regardless of the sensor positioning was observed. The luminescence decay times were evaluated as described above.

**Figure 7.**
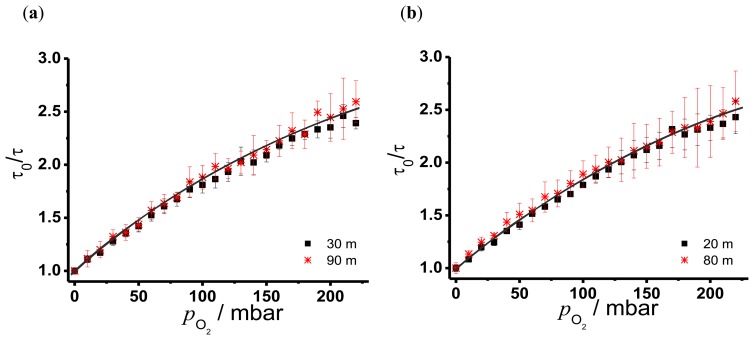
Stern-Volmer plots of TP in silicone (**a**) measured with the branched fiber model (**b**) the single linear fiber model.

In Figure 7 the resulting Stern-Volmer plots for the branched fiber model (Figure 7(a)) and the single linear fiber model (Figure 7(b)) are shown. The larger experimental uncertainties of the second sensor points in Figure 7 are due to the fact that the signal intensities were significantly lower at the second sensor points in both fiber models. These graphs indicate a nonlinear behaviour due to different microenvironments of the sensor molecule in the matrix. The distribution of sensor dye molecules in solid matrices like polymer blends can be divided in accessible and not accessible for the quencher molecule. This leads to an additional term *f* in the Stern-Volmer equation representing the part of the sensor molecules, whose luminescence was quenched by oxygen [26]. The modified Stern-Volmer equation can be written as:

(10) $$\frac{\tau }{\tau_{0}}=\frac{f}{1+K_{\text{SV}}\times p_{O_{2}}}+(1-f)$$

Fitting Equation (9) to the data of Figure 7 results in values for *f* and Stern-Volmer constants that are shown in Table 1.

For all cases the fraction of the quenchable sensor molecules in the matrix could be determined to be 80 %, which is in good agreement with literature data [4]. As expected, the determined *K*_SV_ values using different positions and fiber arrangements show no significant difference. Stern-Volmer constants for oxygen sensing with polycyclic aromatic hydrocarbons (PAHs) in silicone rubber range from 43 × 10^−4^ mbar^−1^ for pyrene to 6.5 × 10^−4^ mbar^−1^ for perylene [6]. TP shows higher *K*_SV_ values (up to a factor of twenty) indicating a higher sensitivity and accuracy for oxygen. Common oxygen sensor dyes like metal porphyrins have Stern-Volmer constants which are larger (7.5 mbar^−1^ for a Pd-metal complex covalently bonded on polymer chain [5]), indicating a greater sensitivity for oxygen quenching. On the other hand the long decay time of such metal porphyrins make them impracticable for the combination with OTDR. The *LOD* was determined for the concentration of oxygen in water. For that the oxygen content in water was calculated with the Henry constant (0.0013 mol/kgbar) [27] and a linear fit of the concentration range between 0 and 100 mbar was performed leading to the *LOD* values in Table 1. The uncertainty of the blank sample *σ* of the first sensor in the branched fiber arrangement was fortuitously low, resulting in a smaller *LOD*. But this is not representative for our oxygen sensor. Therefore, *LODs* around 2 × 10^−5^ M are considered to be typical. The oxygen concentration in water for a partial pressure of 220 mbar is nearly 0.3×10^−3^ M indicating that our sensor is feasible for the mid concentration range of dissolved oxygen in water. With optical sensors specifically optimized for detection of low oxygen concentration *LODs* below 10^−11^ M can be achieved [1].

With the data in Figure 6 the maximal fiber length for sensor application with our experimental setup can be estimated to be 90 m. To increase the maximal fiber length for sensor application the intensity and the total amount of counts at the remote sensor position has to be increased. The measured signals at the last sensor position for both fiber models do not reach 5,000 counts, and for an exact data evaluation the intensity should be around 1,000 counts. Especially for the evanescence wave application the signal intensities of the sensor dye at high oxygen partial pressures are just sufficient for data evaluation. To increase the signal intensity, the dye concentration, the number of measurements (*N*) or the laser pulse power can be increased. The first two possibilities have only a small effect on the total counts at a sensor position. The amount of fluorescence light, which is coupled into the fiber and reaches the detector depends directly on the recapture factor *S*, which is 0.009 for the fiber we used (Equation (4)). Hence, only one percent of the total fluorescence light is usable for the measurements. Furthermore a higher dye concentration will lead to crystallisation of the sensor dye molecules in silicone and therefore to a decreased fluorescence intensity. Increasing *N* results in an increase of the signal-to-noise ratio according to the factor $\sqrt{N}$, leading to inappropriately long measurement time. An increase of the laser pulse power results in a strong reflection of the optical components and leads to a saturation of the detector. Hence an increase of fluorescence signal at the remote sensor points is not possible with this experimental setup. An increase of the maximal fiber length for sensor application is only possible when using a fiber with a lower attenuation coefficient at 355 nm and a higher *NA* and thus a higher *S*.

## 4. Conclusions/Outlook

We have demonstrated the feasibility of combining the technique of optical time domain reflectometry with optical oxygen sensors. For this we established a novel oxygen sensor dye and built a reflectometer for the UV range. The sensor dye and the reflectometer have been characterized with respect to their performance in optical oxygen sensing. The Stern-Volmer plots show a nonlinear behavior and the determined values of *K*_SV_ are in good agreement with literature. The Stern-Volmer values and the LOD indicate that the combination of optical time domain reflectometry and TP for quasi distributed optical oxygen sensing is feasible for the mid concentration range of oxygen in water. The maximal length of the fiber for OTDR sensor applications is approximately 90 m, while using a sensor dye with a decay time of 80 ns (attenuation dead zone of nearly 60 m) results in maximal number of sensor points of two.

## Figures and Tables

**Table 1.** Determined values for f and *K*_SV_ of branched fiber model and of evanescence wave application Limit of Detection (*LOD*) were determined by linear fit of 0 mbar-100 mbar, calculating with Henry constant (0.0013 mol/kg·bar [27]) resulting *LOD* of oxygen in water in mol/L.

	**Branched Fiber Arrangement**		**Evanescent Wave Application**	
		
	**30 m**	**90 m**	**20 m**	**80 m**
***f***	0.8 ± 0.1	0.8 ± 0.1	0.8 ± 0.1	0.8 ± 0.1
***K*_SV_/mbar**^−^**^1^**	0.014 ± 0.003	0.013 ± 0.002	0.012 ± 0.002	0.014 ± 0.002
*LOD*/*10^−^**^5^M (H_2_O)**	0.3 ± 0.2	1.5 ± 0.2	2 ± 0.5	2.3 ± 0.2
